# Synthesis, Crystal, and Electronic Structure of (HpipeH_2_)_2_[Sb_2_I_10_](I_2_), with I_2_ Molecules Linking Sb_2_X_10_ Dimers into a Polymeric Anion: A Strategy for Optimizing a Hybrid Compound’s Band Gap

**DOI:** 10.3390/ijms24032201

**Published:** 2023-01-22

**Authors:** Andrey V. Bykov, Tatiana A. Shestimerova, Mikhail A. Bykov, Liubov A. Osminkina, Alexey N. Kuznetsov, Victoria E. Gontcharenko, Andrei V. Shevelkov

**Affiliations:** 1 Department of Chemistry, Lomonosov Moscow State University, 119991 Moscow, Russia; 2 Physics Department, Lomonosov Moscow State University, 119991 Moscow, Russia; 3 P.N. Lebedev Physical Institute of the Russian Academy of Sciences, 119991 Moscow, Russia

**Keywords:** hybrid organic–inorganic compounds, halometallates, crystal structure, supramolecular ensemble, antimony, polyiodides, electronic structure, band gap

## Abstract

In searching for a tool for optimizing the band gap of a hybrid compound capable of serving as a light-harvesting material in lead-free photovoltaics, we synthesized a new polyiodoantimonate (HpipeH_2_)_2_[Sb_2_I_10_](I_2_) and analyzed its crystal and electronic structure by application of X-ray crystal structure analysis, Raman and diffuse reflectance spectroscopies, and quantum chemical calculations. It was demonstrated that I_2_ molecules link Sb_2_I_10_ edge-sharing octahedra into zig-zag chains, whereas the organic cations link inorganic anionic chains into a 3D structure featuring a complex pattern of covalent bonds and non-covalent interactions. Overall, these features provide the background for forming the electronic structure with a narrow band gap of 1.41 eV, therefore being a versatile tool for optimizing the band gap of a potential light-harvesting hybrid compound.

## 1. Introduction

The discovery of superior light-harvesting properties of inorganic and hybrid haloplumbates that enabled a 25% photovoltaic efficiency of solar cells have triggered great interest in these compounds and led to their meticulous investigation [1]. However, toxicity of lead derivatives raises concerns about potential applications of materials based on haloplumbates. For this reason, other halometallates are now receiving more attention in striving to find non-toxic materials to replace hazardous lead compounds. An obvious scenario of choosing tin instead of lead did not lead to success. Tin in the +2 oxidation state is not stable in halogenides due to its propensity to be oxidized in air and to be disproportionate in an inert atmosphere [2,3,4]. Moreover, tin compounds are also toxic, albeit less than those of lead. The choice of other cations with *s*^2^ lone pairs is limited to Sb3+ and Bi3+, both being not toxic and showing properties favorable for light harvesting. Literature shows that these two cations form a variety of compounds with halogens, demonstrating diverse crystal structures [5,6,7].

Up to now, no significant breakthrough has been achieved in reaching reasonable light-harvesting properties for bismuth and antimony halide derivatives. Their photovoltaic efficiency is as low as 4% [8,9]. At the same time, both Sb^3+^ and Bi^3+^ demonstrate properties that are favorable for solar light harvesting. They have polarizable electron shells, active lone electron pairs, and strong spin-orbit coupling. Moreover, their compounds are not toxic. The main drawback of haloantimonates and halobismuthates that prevents their use is that they demonstrate band gaps exceeding 2 eV, which are too wide for applications in photovoltaics [5,7,10,11,12]. To make them good candidates for light harvesting, it is necessary to narrow the gap down to about 1.5 eV. Therefore, band gap engineering comes to the forefront of developing light-harvesting lead-free halometallates.

Basically, there are three tools for narrowing the band gap. As a rule, the band gap is wide when 0D anions form the anionic substructure of halometallates. This is observed for various compounds based on MX_6_^3−^ anions (M = Sb, Bi; X = Cl, Br, I) [5,7,10,12]. When the octahedra condense into 1D or 2D arrays, the band gap narrows. KBiI_4_·H_2_O and (C_15_H_14_N_4_)BiBr_5_·H_2_O with one-dimensional anions of different compositions and structures provide examples of band gaps of 1.70–1.75 eV [13,14]. The second tool is the use of multiple weak interactions, such as hydrogen bonds and secondary X···X interactions (X = Cl, Br, I). It was shown that such interactions contribute to narrowing the band gap, even in the case of 0D anionic substructure [15]. However, the efficiency of this tool is considerably lower, and typical band gaps of 1.9–1.95 eV were reported [16]. Finally, halogen molecules or polyhalide anions can be introduced into an anionic substructure. They interact with halometallate fragments, linking them into an extended array, and contribute to the vicinity of the Fermi level, making the gap significantly narrower [17]. In such cases, band gaps in the range of 1.25–1.65 eV can be achieved [17,18,19,20]. The latter approach was mainly used for lead and bismuth complex iodides [20,21]. In this research, we expand it onto antimony(III) derivatives.

In the oxidation state of +3, antimony forms many complex halides with a variety of crystal structures. The simplest structures contain stand-alone SbX_6_^3−^ distorted octahedral anions and other simple anions with lower coordination numbers [22,23]. Octahedral anions can join into oligomers, with the number of Sb^3+^ centers ranging from 2 to 10 [5,24,25,26]. Further polymerization leads to chain-like structures of different topology [26,27] and finally to layered structures exemplified by A_3_Sb_2_X_9_ (A = K, Rb, Cs, NH_4_; X = Cl, Br, I) [28,29]. In this work, we have chosen antimony iodide and iodine because polyiodides show greater chemical and thermal stability than other polyhalides and because Sb(III) is stable against oxidation in the presence of iodine. As for the organic cationic part, the choice of homopiperazine (1,4-diazacycloheptane, Hpipe) is explained by the ability of its doubly protonated cation to function as a structure-directed template. We previously showed that the (HpipeH_2_)^2+^ cation forms four hydrogen bonds in various polyiodides and halobismuthates, leading to hybrid compounds with the enhanced stability [30,31].

In this paper, we present a new hybrid compound, (HpipeH_2_)(Sb_2_I_10_)(I_2_), in which I_2_ molecules link Sb_2_I_10_ edge-shared bioctahedra into chains, and discuss its crystal and electronic structure, paying special attention to the role of I_2_ molecules both in forming the anionic substructure and narrowing the band gap.

## 2. Results and Discussion

A facile reaction of SbI_3_ with I_2_, HI, and Hpipe results in formation of black polycrystalline powder with the (HpipeH_2_)(Sb_2_I_10_)(I_2_) composition (Figure S1). The compound is stable in moist air and decomposes only upon heating to 130 °C, releasing one equivalent of I_2_ at the first step.

**Figure 1:**
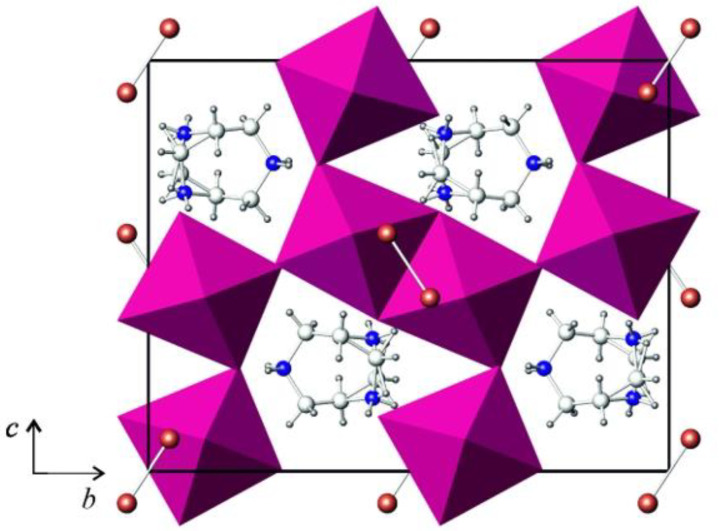
Projection of the crystal structure of (HpipeH_2_)_2_[Sb_2_I_10_](I_2_) onto the (100) plane. Sb_2_I_10_ octahedra, magenta; iodine, brown; nitrogen, blue; carbon, light grey; hydrogen, dark grey.

The crystal structure of (HpipeH_2_)(Sb_2_I_10_)(I_2_) is composed of the inorganic anionic part, in which distorted Sb_2_I_10_ bioctahedra are linked by I_2_ molecules into zig-zag chains, and HpipeH_2_^2+^ cation balancing the charge (Figure 1). The bioctahedra are distorted; the distances from antimony to terminal iodine atoms lie in the range of 2.80–3.01 Å, whereas those to bridging atoms are 3.12 and 3.34 Å (Table 1). A pronounced distortion of the octahedron is not surprising, as the 5*s*^2^ lone electron pair of Sb^3+^ is stereochemically active and tends to find its own place in the coordination environment of antimony(III) [32].

**Figure 2:**
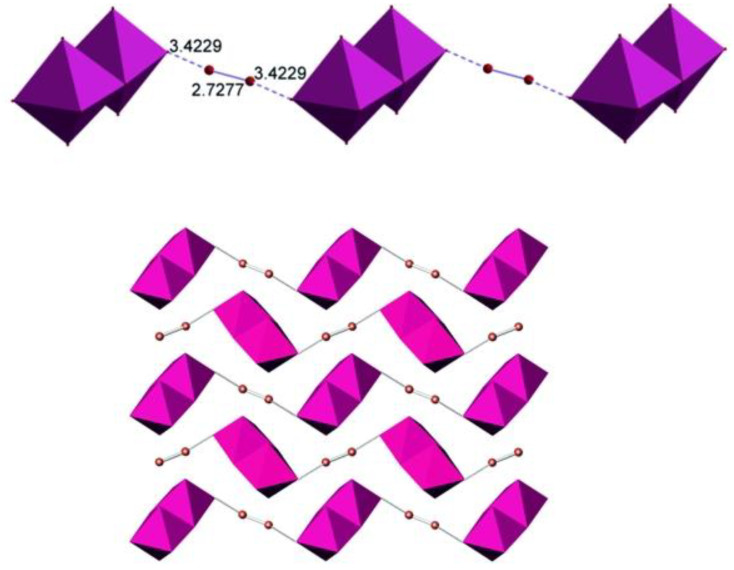
Anionic chain in the crystal structure of (HpipeH_2_)_2_[Sb_2_I_10_](I_2_) (**top**) and the alignment of chains along the *c-*axis (**bottom**). Cations are not shown for clarity. The color code is the same as in Figure 1.

The I_2_ molecules bridge the Sb_2_I_10_ dimers into chains (Figure 2). The I−I distance within the I_2_ moiety is 2.73 Å, which is only 0.02 Å longer than in crystalline iodine [33]. The I···I distance that reflects the interaction of the I_2_ molecule with the iodine atom of the Sb_2_I_10_ bioctahedron is 3.42 Å, with the I···I−I angle of about 168 deg. This distance is shorter than the intralayer distance of 3.50 Å in the crystal structure of iodine. However, such intralayer interactions ensure formation of the grey crystalline solid out of violet I_2_ diatomic gas, altering its properties, which includes the metallic luster, anisotropic conductivity, and the change of the Raman shift of the I−I stretching from 218 to 180 cm^−1^ [34]. A comparison with the literature data shows that the I···I distances in similar crystal structures cover a relatively wide range of distances. For antimony, only two examples with I_2_ molecules interchanging with Sb_2_I_9_ face-shared bioctahedra are known, with the I_2_···I distances of 3.26 and 3.42 Å [35]. More data is available for polyiodoplumbates and polyiodobismuthates. For instance, the I···I interactions of 3.36 and 3.55 Å ensure formation of a 3D anionic substructure out of Pb/I layers [21]. The range of I···I_2_ contacts in bismuth derivatives is even wider, from 3.17 to 3.53 Å [17,20], the former being very short and resembling the longer interatomic distances in various polyiodides [34]. Additionally, they resemble the I···I distances in cluster iodides and polyiodides of transition metals [36,37].

The HpipiH_2_^2+^ cations link the anionic chains into a 3D array by hydrogen bonds. As the symmetry plane intersects the cation, it has two equally probable orientations (Figure 1). Nevertheless, the two orientations are identical in terms of cation–anion interactions. The latter can be described as hydrogen (N)H···I bonds between hydrogen atoms at nitrogen atoms of the cyclic cation with iodine atoms in the vertices of the Sb_2_I_10_ dimers. Each nitrogen atom forms two such bonds; therefore, four (N)H···I bonds are formed by each cation, which means that the cation realizes all its capacity to form hydrogen bonds. As a matter of fact, in various polyiodides and iodobismuthates, HpipeH_2_^2+^ always behaves in a similar fashion in forming four hydrogen bonds, thus serving as a strong structure-directing template [30,31].

In the title compounds, the (N)H···I distances cover the range of 2.76–2.90 Å (Table 2), which is typical for such an interaction type. The same distances were found in various compounds formed by different metals and different organic ammonium cations [30,38,39]. Additionally, there are (C)H···I and longer I···I bonds; however, their involvement into the formation of the 3D structure seems to be limited. The former distances exceed 3 Å, the shortest being 3.09 Å, whereas the latter ones, from 3.77 to 4.00 Å, are only slightly below twice the van-der-Waals radius of iodine [40].

**Figure 3:**
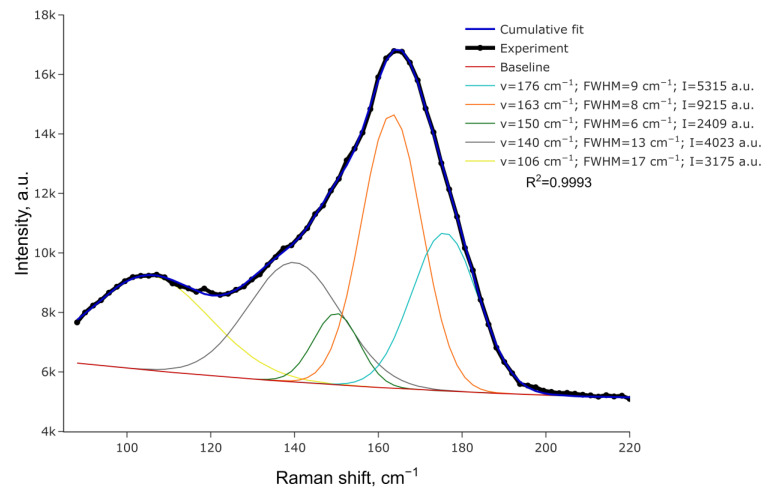
Raman spectrum for (HpipeH_2_)_2_[Sb_2_I_10_](I_2_) and its deconvolution.

Figure 3 portrays the Raman spectrum for (HpipeH_2_)(Sb_2_I_10_)(I_2_). It presents a wide peak near 165 cm^−1^ with asymmetric shoulders. The spectrum can be deconvoluted to reveal five distinct peaks, as shown in Figure 3. The peak at 176 cm^−1^ clearly reflects the I−I stretching of the I_2_ moiety. Compared to solid iodine, where the I−I stretching is observed at 180 cm^−1^ [34], this peak shifts slightly to lower energy, reflecting minor elongation of the I−I bond. Peaks at 163, 150, and 140 cm^−1^ can be assigned to vibrations of Sb−I bonds of different lengths, with the Raman shift increasing, going from longer Sb−I^μ^ separations to shorter Sb−I^t^ interatomic distances (μ—bridging; t—terminal) [41,42]. The peak at 106 cm^−1^ is difficult to attribute unambiguously, as various vibrations can appear at this Raman shift, including Sb_2_I_10_ bending. Additionally, this peak is wider and might as well be an overlap of unresolved close peaks.

**Figure 4:**
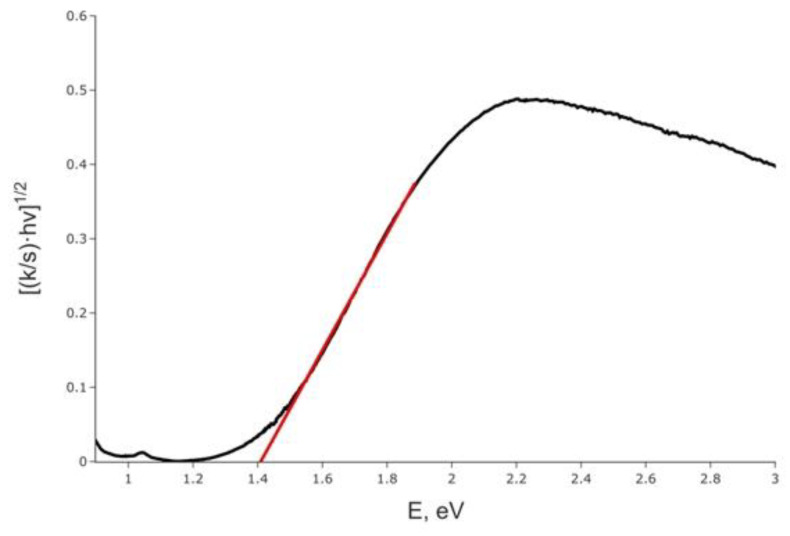
Kubelka–Munk plot for (HpipeH_2_)_2_[Sb_2_I_10_](I_2_), assuming the indirect band gap (black line) and approximation of the band gap (red line).

Optical diffuse reflectance spectrum was measured for the title compound and converted into the absorbance data presented in Figure 4. The Kubelka–Munk transformation was performed assuming the indirect band gap according to the results of calculations (vide infra), and extrapolation of the linear part onto the energy axis results in the gap width of 1.41 eV, which nicely corresponds to the black color of the title compound. Surprisingly, the band gap is almost the same as in recently reported (Me_4_N)_3_{[M_2_I_9_](I_2_)}, 1.40 eV, in which face-shared Sb_2_I_9_ dimers are linked by I_2_ molecules [35]. The latter compound shows a slightly longer I−I covalent bond of 2.74 Å but a slightly shorter I_2_···I separation of 3.26 Å compared to (HpipeH_2_)(Sb_2_I_10_)(I_2_). It looks like a slight deviation in the interatomic distances as well as a different nature of the Sb−I dimer play only a minimal role in altering the optical properties of the polyiodoantimonates.

**Figure 5:**
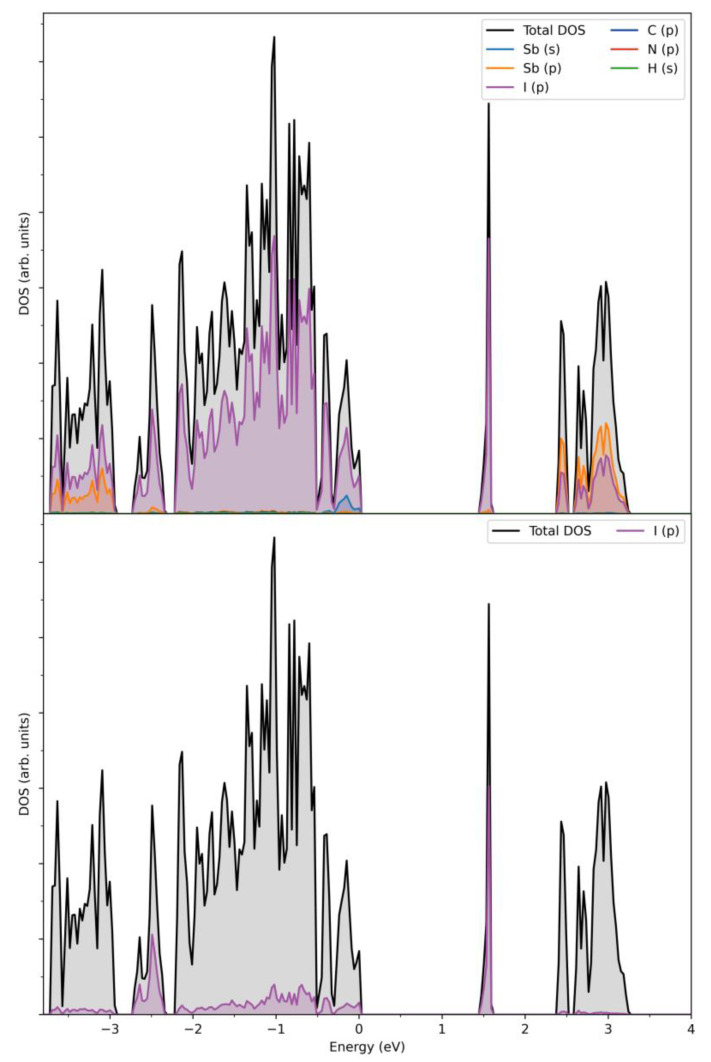
Calculated total and projected densities of states near the Fermi level for (HpipeH_2_)_2_[Sb_2_I_10_](I_2_) (**top**) and projected I_2_ *5p* state (**bottom**). Fermi energy is at zero.

In order to gain deeper insight into the nature of electronic structure on the local and extended scales, density-function calculations (DFT) were performed on the title compound. Total and projected densities of states near the Fermi level (DOS), calculated for an ordered model of the periodic structure of (HpipeH_2_)_2_[Sb_2_I_10_](I_2_), are shown in Figure 5. As is typical for the compounds of this type, a narrow-gap semiconductor, the estimated band gap is 1.45 eV, and the largest contributions to both the valence and conduction band near the Fermi level arise from the I 5*p* states, with Sb 5*p* states also contributing to the bottom of the conduction band and 5*s* to the top of the valence band, albeit to a lesser degree. Sb 5*p* states are almost empty, which indicates that its formal charge is close to +3. The sharp density peak at ca. 1.5 eV is very narrow, indicating highly localized states. It is composed of 5*p* states of the I_2_ moieties and is separated from other states at the bottom of the conduction band by ca. 0.8 eV (Figure 5). It can be viewed as analogous to the in-gap state responsible for the acceptor properties. Therefore, the charge transfer proceeds from the 5*p* states of iodine atoms at the vertices of the Sb_2_I_10_ dimers to the 5*p* states of the I_2_ units.

**Figure 6:**
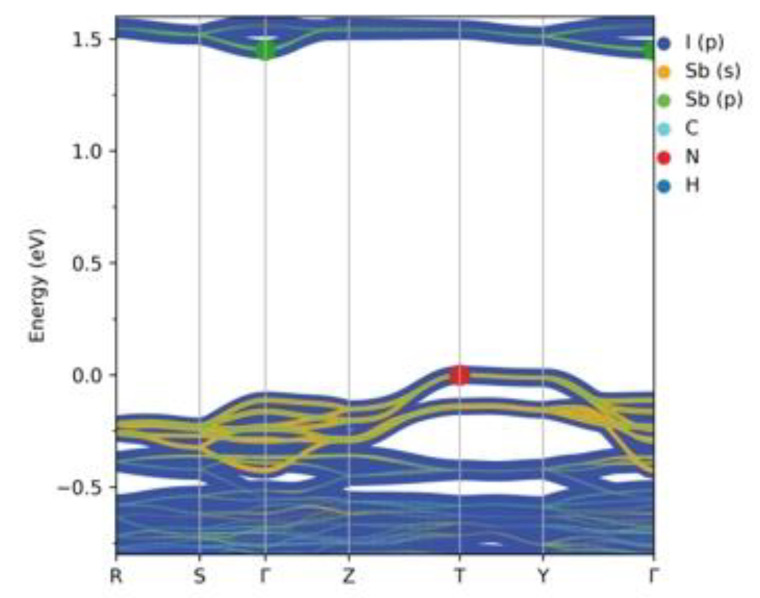
Calculated band structure for (HpipeH_2_)_2_[Sb_2_I_10_](I_2_). Atomic contributions are colored according to the legend. Band thickness is proportional to the size of a contribution. The green dot denotes CBM, while the red dot VBM. Fermi energy is at zero.

Calculated band dispersion along the high-symmetry points (Figure 6) confirms the results of the DOS analysis and shows that (HpipeH_2_)_2_[Sb_2_I_10_](I_2_) is an indirect gap semiconductor with the band gap of 1.45 eV, as was established by the DOS analysis, with valence band maximum (VBM) at the T-point and conduction band minimum (CBM) at the Γ-point.

**Figure 7:**
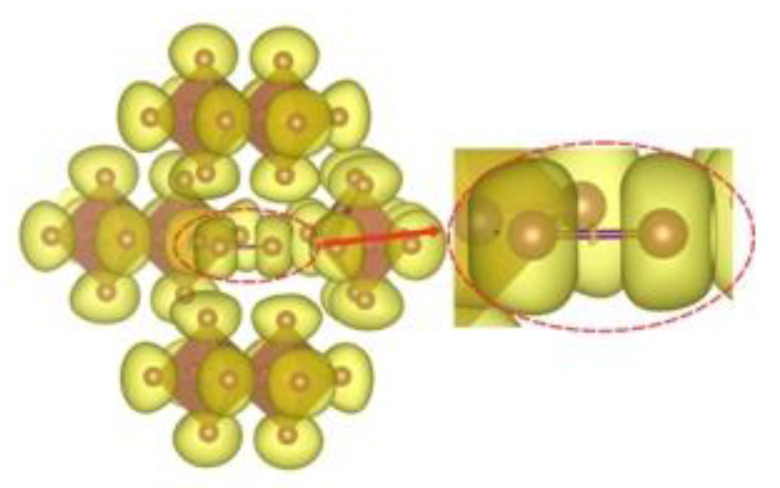
ELF isosurface (yellow, *η* = 0.605) for the [Sb_2_I_10_^4−^]_4_I_2_ cluster. An insert magnifies a single non-atom centered attractor between the two I atoms of the I_2_ unit in the center of the figure.

**Figure 8:**
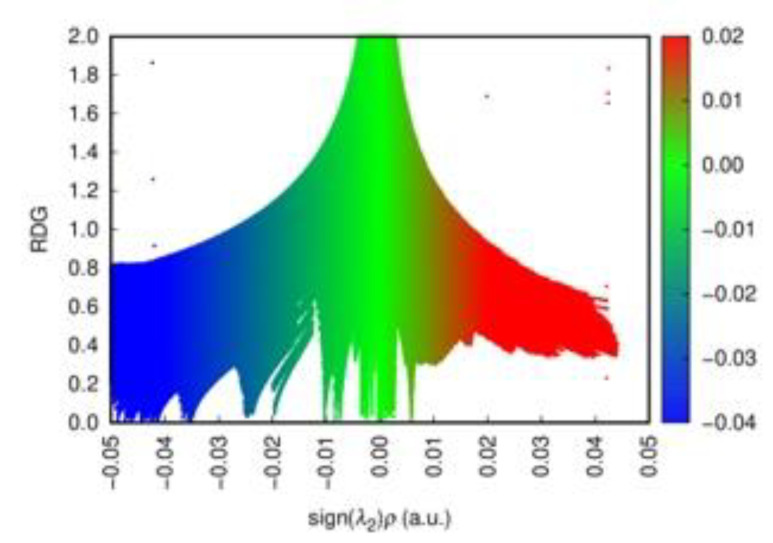
The scatter diagram for the sign(λ^2^)*ρ*(r) plot for the [Sb_2_I_10_^4−^]_4_I_2_ cluster. Strong NCIs are colored in blue, weak ones in green, and non-bonding interactions in red. The RGB color scale to the right of the plot corresponds to the change from strong to weak to non-bonding NCI within the calculated range of −0.04 a.u. < sign(λ^2^)*ρ*(r) < 0.02 a.u.

In order to gain an insight into the bonding pattern in the title compound, the electron-localization-function (ELF) topology was calculated and analyzed for the 3D case, as well as for the ionic clusters cut from the periodic structure. The latter can provide better detail, particularly when studying weaker interactions, as well as be more convenient to study in a relatively close-packed structure with many interacting fragments, which creates a rather busy and somewhat cluttered picture in the case of 3D structure. The results demonstrate that the I_2_ unit is a neutral iodine molecule with a covalent bond between the iodine atoms (Figure 7). No covalency is observed between the I_2_ units and [Sb_2_I_10_^4−^] units, as well as between the latter. This agrees very well with the highly localized nature of the 5*p*-states of the I_2_ moieties. Non-covalent-interaction (NCI) analysis based on the reduced-density-gradient (RDG) method [43] allows us to partition the space into regions with relatively strong attraction and weak interaction, which are differentiated by the electron density values (denoted *ρ*(r))—where small values correspond to weak interactions (e.g., van der Waals, dispersive, etc.) and intermediate–to-strong NCI (e.g., hydrogen bonds, halogen bonds, etc.). Negative sign(λ^2^)*ρ*(r) corresponds to attraction and its positive to repulsion. The conventional color-coding of the NCI is based on the values of sign(λ^2^)*ρ*(r), which characterize the strength of the interaction: strong NCI near −0.05 a.u. are colored in blue; weak ones near zero in green; and the brighter weaker ones and non-bonding interactions (above 0.01 a.u.), such as steric repulsion, in red. (For reference, see Figure 8, where the RGB color scale is set to the range of −0.04 a.u. < sign(λ^2^)*ρ*(r) < 0.02 a.u.) The “tails” of the plot pointing downwards mark specific NC interactions.

**Figure 9:**
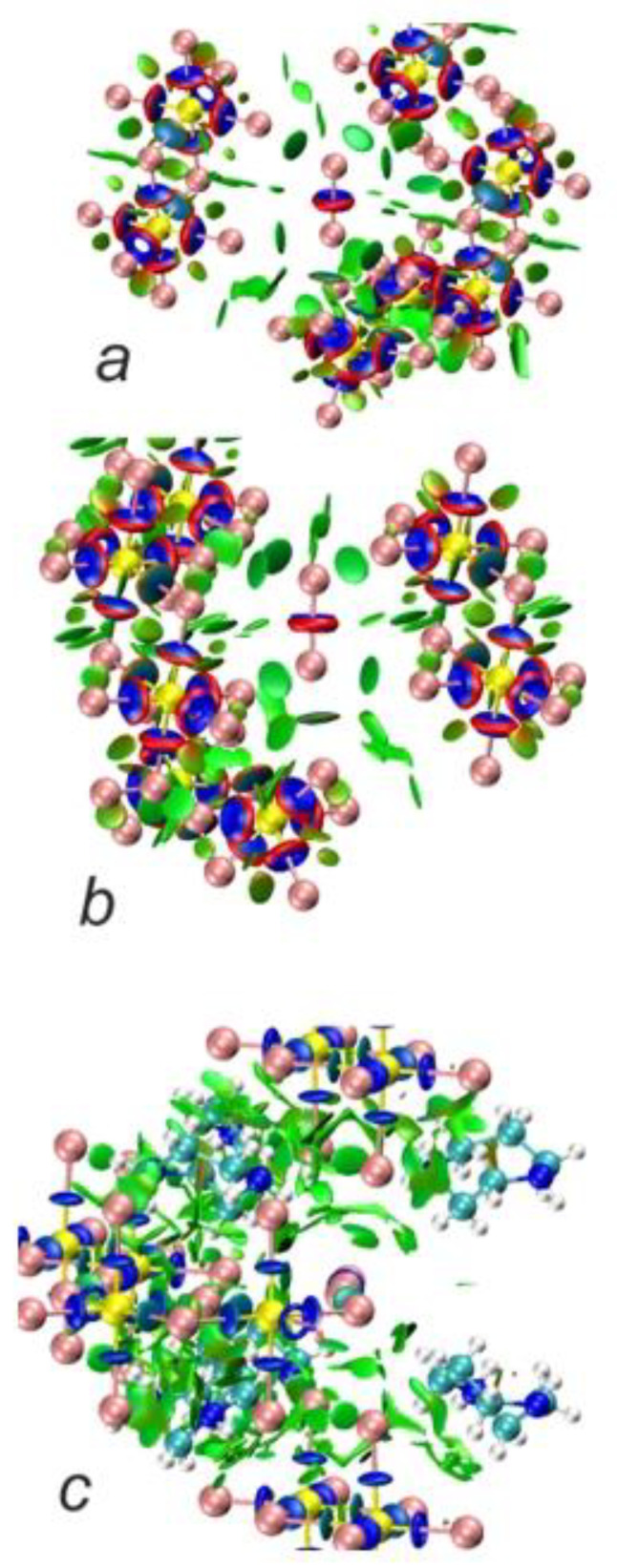
NCI plots for [Sb_2_I_10_^4−^]_4_I_2_ cluster (**a**,**b**; RDG isosurface at 0.5) and (C_5_H_13_N_2_^2+^)_6_[Sb_2_I_10_^4−^]_4_I_2_ cluster (**c**, RDG isosurface at 0.35). Blue discs denote ‘around bond’ areas; green discs and areas denote non-covalent interactions (NCI).

Cation-free NCI plots confirm that the anionic part of the structure consists of the [Sb_2_I_10_^4−^] units with Sb−I bonds showing covalency, as evidenced by blue ‘around bond’ rings surrounded by typical narrow red bands corresponding to electron repulsion regions. The same is observed for the I_2_ molecule embedded in the framework. There is clearly no covalency between I_2_ and anions, and these units are only connected via the NCI. As seen from Figure 9a,b, there is one slightly stronger interaction between I_2_ and [Sb_2_I_10_^4−^]_4_, seen as a blueish green disc, that corresponds to the shortest distance between these units of ca. 3.42 Å (ca. −0.025 a.u. on the interaction scale, according to Figure 8), while the other vertices at ca. 3.99 Å show slightly weaker NCI (where the difference in *ρ*(r) between these two, according to the RDG distribution, is ca. 0.01–0.015 a.u.). Nevertheless, these interactions are clearly of the same nature and fall firmly into the NCI category. Also, there are even weaker NCIs between the iodine atoms of the [Sb_2_I_10_^4-^] units. When we add HpipeH_2_ cations to the picture, we clearly observe weak H···I interactions between terminal iodine and hydrogen atoms; however, based on their *ρ*(r) values, they are not strong enough to be considered proper hydrogen bonds, as the latter are fairly strong and should be at least on par with halogen bonds and be easy to separate from weaker dispersive interactions.

To summarize our analysis, we can establish the structure of the compound as a proper salt, built from the C_5_H_13_N_2_^2+^ cations and [Sb_2_I_10_^4−^] anions, with neutral I_2_ units embedded into the structure and linking the anions. This picture is rather similar to what we previously observed for (NH_4_)_7_Bi_3_I_16_(I_2_)_0.5_·4.5H_2_O [17]. However, despite the general similarity, there is a difference between these cases, i.e., shorter I···I distances between the I_2_ units and BiI_6_ units in the case of the latter compound of ca. 3.17 Å, as compared to ca. 3.42 Å for the Sb-containing compound, which apparently results in higher *p*-state localization in the latter and in a somewhat weaker interaction between the units, of which the most obvious evidence, according to the DOS plots, is an increase in the band gap (ca. 1.2 for the Bi-containing compound vs ca. 1.5 for the Sb-containing one). Thus, while it is probably still possible to call the anionic part of (HpipeH_2_)_2_[Sb_2_I_10_](I_2_) some kind of polymer chain based on the evidence of the stronger NCI between I_2_ and [Sb_2_I_10_^4−^] than between these and any other fragments of the structure, the latter appears to be somewhat weaker than the interactions between I_2_ and BiI_6_ in (NH_4_)_7_Bi_3_I_16_(I_2_)_0.5_·4.5H_2_O [17]. Moreover, whereas the charge transfer in the latter compound occurs from the 5*p* states originated from I_2_ units to the empty 6*p* states of bismuth, in the title compound, the charge transfer proceeds from the 5*p* states of iodine atoms in the vertices of Sb_2_I_10_ dimers to the in-gap states forms by the 5*p* orbitals of the I_2_ moieties.

## 3. Materials and Methods

**Synthesis.** Homopiperazine C_5_N_2_H_12_ (98%, Acros Organics, Geel, Belgium), SbI_3_ (pure, Reakhim, Staraya Kupavna, Russia), and iodine I_2_ (pure, MNT, Moscow, Russia) were used as received. Hydroiodic acid (57%) was synthesized by the procedures described elsewhere [13]. For the synthesis of the title compound, a solution of 0.2886 g (0.574 mmol) of SbI_3_ and 0.1081 g (0.426 mmol) of I_2_ in 20% of HI (13 mL) was added to a solution of 0.0557 g (0.551 mmol) of homopiperazine in the same acid (3 mL). Black crystals formed within a two-week period and were filtered off under vacuum and dried in air at −5 °C.

**Powder X-ray diffraction analysis** (PXRD) was performed on an Imaging Plate Guinier Camera (Huber G670, Cu-Kα1 radiation, λ = 1.540598 Å, Rimsting, Germany). For data collection, crystals were finely crushed in an agate mortar, and the resulting powder was fixed on a holder between two X-ray amorphous films (Figure S2).

**Thermal analysis.** Thermogravimetric analysis was performed using a NETZSCH 209 F1 Libra thermobalance (NETZSCH, Selb, Germany). Samples were heated in alumina crucibles under dry nitrogen flow up to 623 K with the ramp rate of 5 K·min^−1^. The NETZSCH Proteus Thermal Analysis program was used for the data processing. The mass loss at the first stage above 130 °C is 12.52% (−I_2_, 12.88%), see Figure S3.

**Crystal structure determination**. Well-shaped single crystals of the title compound were selected from the reaction product. Single-crystal diffraction data were measured using a Bruker D8 Quest diffractometer (Bruker, Karlsruhe, Germany) equipped with a CMOS detector (MoKα, λ = 0.71073 Å, graphite monochromator) at 120 K [44]. Data were corrected for absorption effects using semiempirical methods implemented in SADABS (2016/2) [45]. The crystal structure was solved by the intrinsic phase methods, which gave the positions of all iodine and antimony atoms. Difference Fourier syntheses in SHELXT (SHELXL, 2018/3) gave the positions of all nitrogen and carbon atoms [46]. Hydrogen atoms on carbon and nitrogen atoms were refined using riding models. The crystal structure was refined by anisotropic approximations of the atomic displacement parameters for all atoms except hydrogens. Isotropic atomic displacement parameters were restricted to 1.2 times for the carbon and nitrogen hydrogens and their respective parent atom. Experimental and crystallographic information is given in Table 3. Further details of the crystal structures may be obtained from the Cambridge Crystallographic Data Centre by quoting the CCDC number 2231705.

**Optical spectroscopy.** Optical diffuse reflectance spectra were recorded using a Perkin-Elmer Lambda 950 UV–visible spectrometer (Perkin-Elmer, Waltham, MA, USA) with an attached diffuse reflectance accessory. Measurements were performed at 298 K in the spectral range of 250–1000 nm, with a scanning rate of 2 nm/s using a finely ground polycrystalline sample. The data were transformed into absorbance using the Kubelka–Munk method and plotted as [(*k*/*s*)·*h*υ]^1/2^ against *h*υ, where *k* is the absorption coefficient, *s* is the scattering coefficient, and *h* is the Planck constant [47]. Optical band gap, *Eg*, was approximated by extrapolation to *k* = 0.

**Raman spectroscopy.** Raman micro-spectroscopy data were acquired for **(**HpipeH_2_)_2_{[Sb_2_I_10_](I_2_)} using a confocal Raman microscope Confotec™ MR350 system, with a laser excitation at 633 nm wavelength and 0.5 mW power. Processing of the Raman spectrum was carried out with the RamPy Python library [48]. Baseline was approximated by the third-degree polynomial, and vibration bands were fitted with Gaussian functions; then error minimization of the cumulative fit was performed using the least-squares algorithm with 100 steps.

**Computational details**. DFT calculations on the periodic structure of C_5_H_13_N_2_SbI_6_ were performed using the projector augmented-wave method (PAW) as implemented in the Vienna Ab initio Simulation Package (VASP) [49,50]. The r^2^SCAN exchange-correlation functional [51,52] of the meta-GGA type combined with the RVV10 correction for dispersive interactions [53] was used for the calculations, with a Brillouin zone sampling using a Monkhorst-Pack [54] grid of 8 × 8 × 8 k-points. Experimental structural information was used for the modeling, with the ordering of C and N confined to a single orientation of the C_5_H_13_N_2_^2+^ cations. The energy cutoff was set to 500 eV, and the energy-convergence criterion was at 10^−5^ eV. Convergence towards the k-point set and energy was checked. DFT calculations on the molecular fragments was performed in two ways: (i) on the anionic cluster consisting of four [Sb_2_I_10_]^4−^ units surrounding the I_2_ unit and (ii) on the same cluster with six C_5_H_13_N_2_^2+^ cations added. In both cases, experimental structure was used to construct clusters, and the calculations were performed using the r^2^SCAN-3c method [55] and ma-def2-TZVP basis sets (28 *e* core ECP28MDF) [56] utilizing the Orca 5.0.3 package [57,58]. ELF [59,60,61] and RDG analyses were performed using the Multiwfn 3.8 package [62]. ELF topology analysis was performed using the VESTA package [63], and NCI plots were done using VMD 1.9.4 [64]. DOS and band plots were produced using the SUMO 2.3.5 package [65].

## 4. Conclusions

We synthesized a hybrid compound (HpipeH_2_)(Sb_2_I_10_)(I_2_), which is composed of Sb_2_I_10_ edge-shared bioctahedra linked by I_2_ molecules into zig-zag chains aligned along the *c-*axis of the unit cell and alternating with the HpipeH_2_^2+^ charge-balancing cations. The title compound is only the third example of polyiodoantimonates after two recently published compounds containing Sb_2_I_9_ face-shared octahedra linked by the same I_2_ molecules [35]. The electronic structure of (HpipeH_2_)(Sb_2_I_10_)(I_2_) assessed by Raman and diffuse reflection spectroscopies and DFT analysis revealed details of the band structure and the pattern of covalent and non-covalent interactions within the inorganic anion and in the cation-anion system. It is found that the indirect band gap (experimental, 1.41 eV; calculated, 1.45 eV) is constructed by the iodine 5*p* states at the top of the valence band and antimony and iodine 5*p* states at the bottom of the conduction band. The analysis of the atomic contributions enabled us to conclude that the charge transfer occurs mainly from 5*p* states of I atoms at the vertices of the Sb_2_I_10_ dimers to the 5*p* states of I_2_ moieties. The analysis of the electronic localization function topology showed that covalent Sb−I and I−I bonds exist within the Sb_2_I_10_ and I_2_ moieties, respectively, whereas the non-covalent interaction analysis revealed stronger I_2_···I interactions, which can be regarded as halogen bonds, along with weaker (N)H···I hydrogen bonds between cations and anions and I···I interaction between the vertices of the Sb_2_I_10_ bioctahedra. The results of this research show that the inclusion of the I_2_ molecule into the anionic part of hybrid compounds with antimony–iodine anions is a facile synthetic procedure that leads to narrowing the band gap by exploiting multiple weak interactions, ensuring the formation of 3D structures.

## Figures and Tables

**Table 1:** Selected interatomic distances and angles in the crystal structures of (C_5_N_2_H_14_)_2_[Sb_2_I_10_](I_2_).

Atoms	Distance, Å	Atoms	Angle, °
Sb1–I1 ^μ^ Sb1–I1 ^μ^ Sb1–I2 ^t^ Sb1–I3 ^t^ Sb1–I4 ^t^ I5–I5 I5···I3 I2···I3 I2···I3	3.1186(8) 3.3420(9) 2.8032(8) 2.9398(8) 3.0058(4) 2.7281(14) 3.4229(9) 3.7747(8) 3.9972(8)	I5—I5—I3 Sb1—I3—I5 I1—Sb1—I1 I4—Sb1—I4 I3—Sb1—I1 I2—Sb1—I1 I4—Sb1—I1	167.65(4) 111.99(2) 87.15(2) 173.92(3) 176.92(3) 175.78(3) 91.199(14)

μ—bridging; t—terminal

**Table 2:** Hydrogen bonding in the crystal structure of (C_5_N_2_H_14_)_2_[Sb_2_I_10_](I_2_).

D–H···A D	d(D-H), Å	d(H…A), Å	d(H…A), Å	∠(D-H…A), °
N1–H3···I3	0.91	2.86	3.747(3)	164.0
N1–H3···I3	0.91	2.86	3.747(3)	164.0
N2–H2A···I1	0.90	2.75	3.502(11)	141.4
N2–H2B···I4	0.90	2.90	3.604(10)	136.1
C1–H1B···I4	0.99	3.06	3.803(7)	133.3
C2–H2AA···I1	0.96	3.09	3.947(8)	149.6

**Table 3:** Crystallographic data for (C_5_N_2_H_14_)_2_[Sb_2_I_10_]I_2_ at 110 K.

Empirical Formula	(HpipeH_2_)_2_{[Sb_2_I_10_](I_2_)}
Molecular weight	984.32
Temperature (K)	100(2)
Crystal system	Orthorhombic
Space group	*C*mce
Cell parameter, *a, b, c* [Å]	13.6043(4)
	18.4176(5)
	14.5100(4)
Volume [Å^3^]	3635.60(18)
Z	8
Density (calculated) [g cm^−3^]	3.597
Diffractometer	Bruker D8 VENTURE
Radiation, λ [Å]	0.71073 (MoKα)
Data collection range (°)	2.21–28
R_int_	0.0445
μ (MM^−1^)	11.685
R_1_ [F_0_ > 4σF_0_]	0.0312
wR_2_ [F_0_ > 4σF_0_]	0.0662
Goodness-of-fit	1.070
(e/Å^−3^)	1.601/−2.813

## Data Availability

Not applicable.

## Supplementary Materials

The following supporting information can be downloaded at https://www.mdpi.com/article/10.3390/ijms24032201/s1.
