# Supplementary material for: Synthesis, Crystal, and Electronic Structure of (HpipeH2)2[Sb2I10](I2), with I2 Molecules Linking Sb2X10 Dimers into a Polymeric Anion: A Strategy for Optimizing a Hybrid Compound’s Band Gap
Source: Int J Mol Sci. 2023 Jan 22;24(3):2201. doi: 10.3390/ijms24032201 (PMC9916808; doi:10.3390/ijms24032201)
Supplement: Supplementary file 1 [file ijms-24-02201-s001.zip › ijms-2145091-supplementary.pdf]

International Journal of *Molecular Sciences*

MDPI

Article

Synthesis, Crystal, and Electronic Structure of $(HpipeH_2)_2[Sb_2I_{10}](I_2)$, with I_2 Molecules Linking Sb_2X_{10} Dimers into a Polymeric Anion: A Strategy for Optimizing a Hybrid Compound's Band Gap

Andrey V. Bykov [1], Tatiana A. Shestimerova [1], Mikhail A. Bykov [1], Liubov A. Osminkina [2], Alexey N. Kuznetsov [1], Victoria E. Gontcharenko [3] and Andrei V. Shevelkov [1,*]

[1] Department of Chemistry, Lomonosov Moscow State University, 119991 Moscow, Russia
[2] Physics Department, Lomonosov Moscow State University, 119991 Moscow, Russia
[3] P.N. Lebedev Physical Institute of the Russian Academy of Sciences, 119991 Moscow, Russia
* Correspondence: shev@inorg.chem.msu.ru

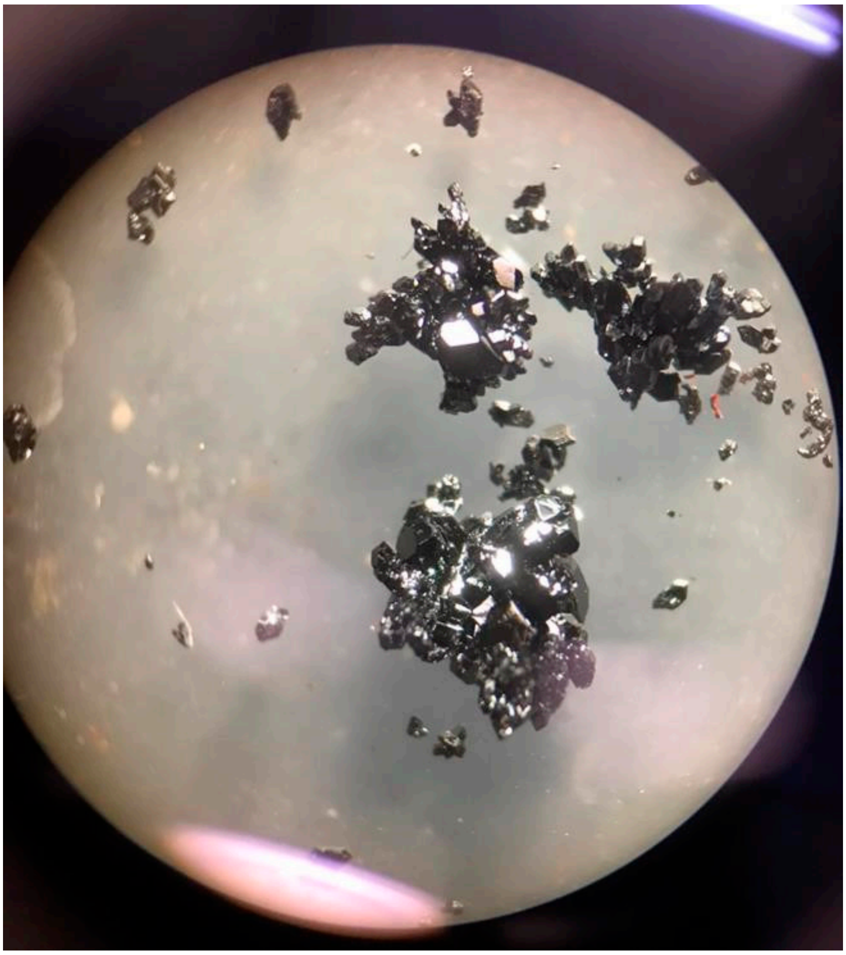

Figure S1. Crystals of the compound $(C_5N_2H_{14})_2[Sb_2I_{10}](I_2)$.

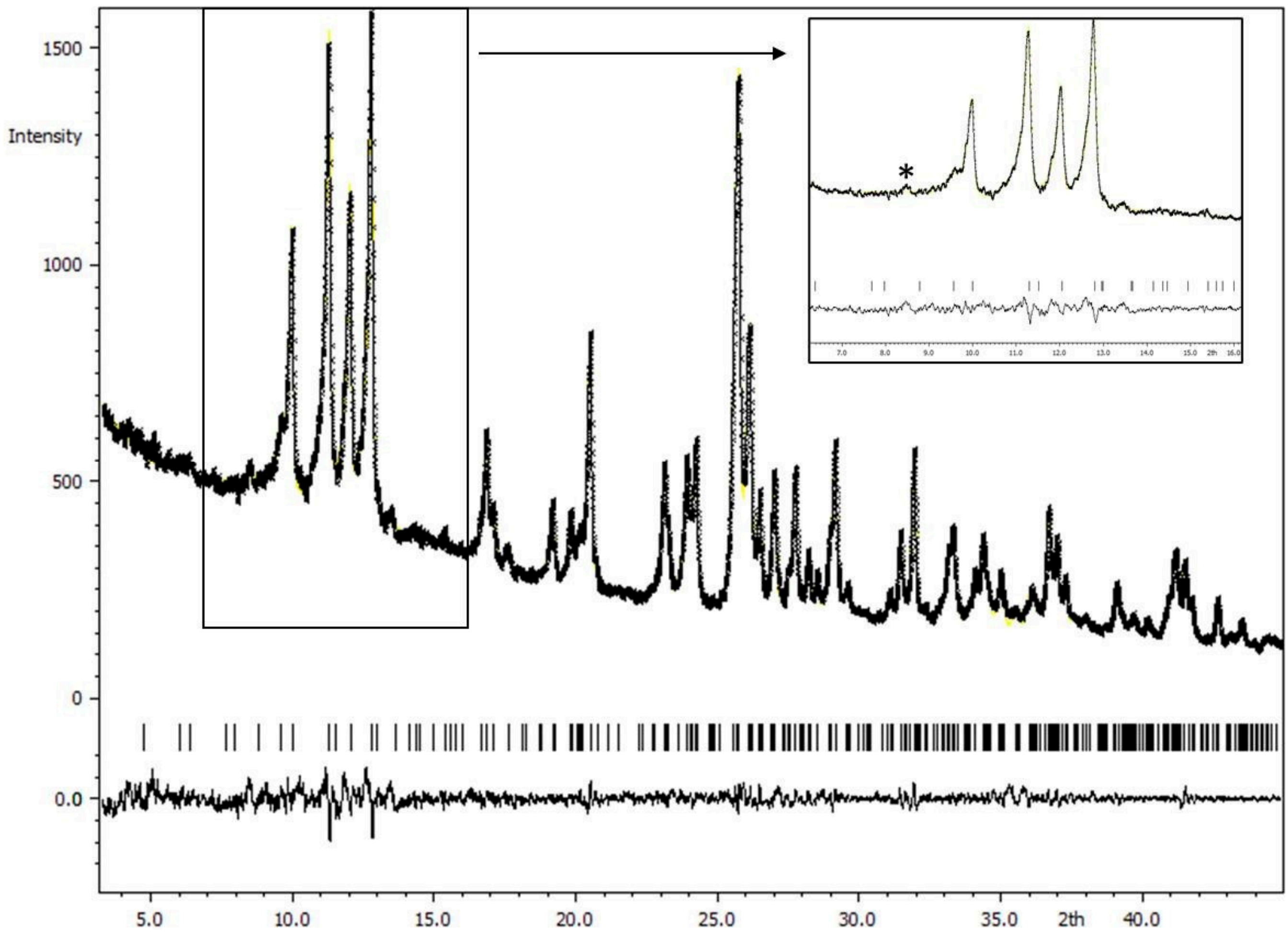

Figure S2. Powder X-ray diffraction pattern and Rietveld refinement for $(C_5N_2H_{14})_2[Sb_2I_{10}](I_2)$. The upper curve represents the experimental diffraction pattern, the ticks show calculated peak positions, and the lower curve is the difference between the experimental and calculated patterns. The most significant area from 4 to 50° 2 is shown. The enlarged fragment of the X-ray diffraction pattern and Rietveld refinement from 6 to 16° 2 is shown as an inset, on which an asterisk indicates the only reflection of the - $(C_5N_2H_{14})_2[Sb_2I_{10}]$ $2H_2O$ admixture (CCDC2190422).

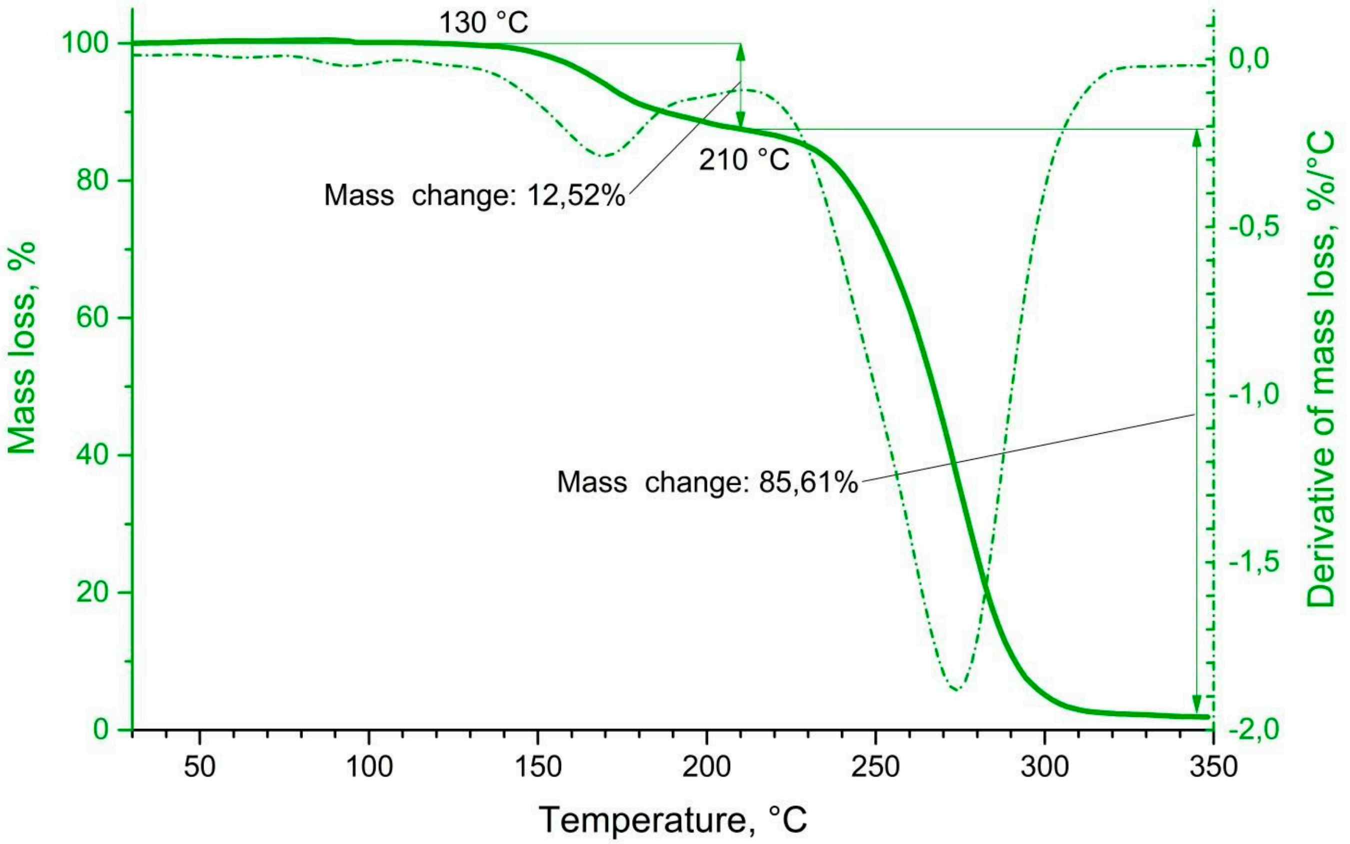

Figure S3. TGA diagram for $(C_5N_2H_{14})_2[Sb_2I_{10}](I_2)$
